# Development of a simplified model and nomogram in preoperative diagnosis of pediatric chronic cholangitis with pancreaticobiliary maljunction using clinical variables and MRI radiomics

**DOI:** 10.1186/s13244-023-01383-z

**Published:** 2023-03-08

**Authors:** Yang Yang, Xin-xian Zhang, Lian Zhao, Jian Wang, Wan-liang Guo

**Affiliations:** 1 Department of Radiology, Children’s Hospital of Soochow University, Suzhou, 215025 China; 2 Department of Radiology, Xuzhou Children’s Hospital, Xuzhou, 221002 China; 3 Pediatric Surgery, Children’s Hospital of Soochow University, Suzhou, 215025 China

**Keywords:** Pancreaticobiliary maljunction, Children, Magnetic resonance imaging, Radiomics, Nomogram

## Abstract

**Objective:**

The aim of this study was to develop a model that combines clinically relevant features with radiomics signature based on magnetic-resonance imaging (MRI) for diagnosis of chronic cholangitis in pancreaticobiliary maljunction (PBM) children.

**Methods:**

A total of 144 subjects from two institutions confirmed PBM were included in this study. Clinical characteristics and MRI features were evaluated to build a clinical model. Radiomics features were extracted from the region of interest manually delineated on T2-weighted imaging. A radiomics signature was developed by the selected radiomics features using the least absolute shrinkage and selection operator and then a radiomics score (Rad-score) was calculated. We constructed a combined model incorporating clinical factors and Rad-score by multivariate logistic regression analysis. The combined model was visualized as a radiomics nomogram to achieve model visualization and provide clinical utility. Receiver operating curve analysis and decision curve analysis (DCA) were used to evaluate the diagnostic performance.

**Results:**

Jaundice, protein plug, and ascites were selected as key clinical variables. Eight radiomics features were combined to construct the radiomics signature. The combined model showed superior predictive performance compared with the clinical model alone (AUC in the training cohort: 0.891 vs. 0.767, the validation cohort: 0.858 vs. 0.731), and the difference was significant (*p* = 0.002, 0.028) in the both cohorts. DCA confirmed the clinical utility of the radiomics nomogram.

**Conclusion:**

The proposed model that combines key clinical variables and radiomics signature is helpful in the diagnosis of chronic cholangitis in PBM children.

**Supplementary Information:**

The online version contains supplementary material available at 10.1186/s13244-023-01383-z.

## Key points

- Conventional imaging modalities were not powerful enough to diagnose chronic cholangitis.
- The radiomics signature based on T2-weighted MR images performed well in diagnosing chronic cholangitis.
- Associating the radiomics signature with clinical factors improved the diagnosis performance of chronic cholangitis.

**Supplementary Information:**

The online version contains supplementary material available at 10.1186/s13244-023-01383-z.

## Introduction

Pancreaticobiliary maljunction (PBM) is a rare congenital anomaly with a reported incidence of 1:1000 in the Asian population, which is 100 to 1000 times higher than in other parts of the world [1, 2]. PBM is characterized by the junction of the pancreatic and biliary ducts outside the duodenal wall [1, 3]. Such an anomaly allows regurgitation between the pancreatic and biliary tract. Higher pressure in the pancreatic duct leads to reflux of pancreatic juice into the bile duct [4]. As a result, PBM is often accompanied by repeated episodes of acute cholangitis, chronic cholangitis, and even cholangiocarcinoma [1, 5]. Chronic cholangitis increases the fragility of the bile duct and adhesion to the surrounding tissues, and thus increase the risk of iatrogenic injury during pancreatic-duct surgery [6] and prolonged surgical duration of complete cyst excision [7]. Preoperative diagnosis of chronic cholangitis in PBM children scheduled for surgery is thus critically important.

In routine clinical practice, ultrasonography (US), computed tomography (CT), and magnetic-resonance cholangiopancreatography (MRCP) comprise the most frequently used noninvasive imaging modalities available in diagnosing cholangitis [8]. MRI-MRCP provides superior contrast resolution and clearly delineates the bile duct without the use of a contrast agent, and thus is the preferred diagnostic modality in the pediatric population [8]. However, interpreting imaging is highly complex and requires vast experience in the assessment of stenosis and dilation of bile ducts, thickened bile duct walls, heterogeneous enhancement of these walls, protein plug, signs of periductal inflammation, and abnormalities of hepatic parenchymal tissue [9–11], and thus is limited in accuracy and sensitivity [9, 10].

Radiomics combined with rapid machine learning (ML) paradigms has been increasingly used recently as a diagnostic tool for many diseases [12]. Radiomics is a high-throughput computational method that unlocks microscale quantitative data hidden within conventional images and offers insight into the heterogeneity of lesions that are unobservable by the naked eye [13, 14]. A previous study of our research group established the feasibility of using radiomics and deep learning to define chronic inflammation of the biliary wall in PBM children [15]. In the current study, we developed a model and a nomogram that combine clinically relevant features with radiomics signature based on T2-weighted MR images for diagnosis of chronic cholangitis in PBM children.

## Materials and methods

This study was approved by the Institutional Review Boards of two participating hospitals. Requirement for informed consent was waived due to the retrospective nature of the study.

### Diagnostic criteria for PBM and chronic cholangitis

PBM was diagnosed preoperatively based on MRCP or CT showing convergence of the pancreatic and bile ducts outside the duodenal wall and abnormally long common channel (> 5 mm), and confirmed by intraoperative cholangiography (IOC) in all cases [5, 16].

Chronic cholangitis was diagnosed based on chronic inflammation of the bile duct wall on pathological examination under local protocol. Features that were considered included hyperemia, edema, inflammatory infiltration, exfoliation of the mucous epithelium, and proliferation of fibrous tissue [17].

### Patients

**Fig. 1:**
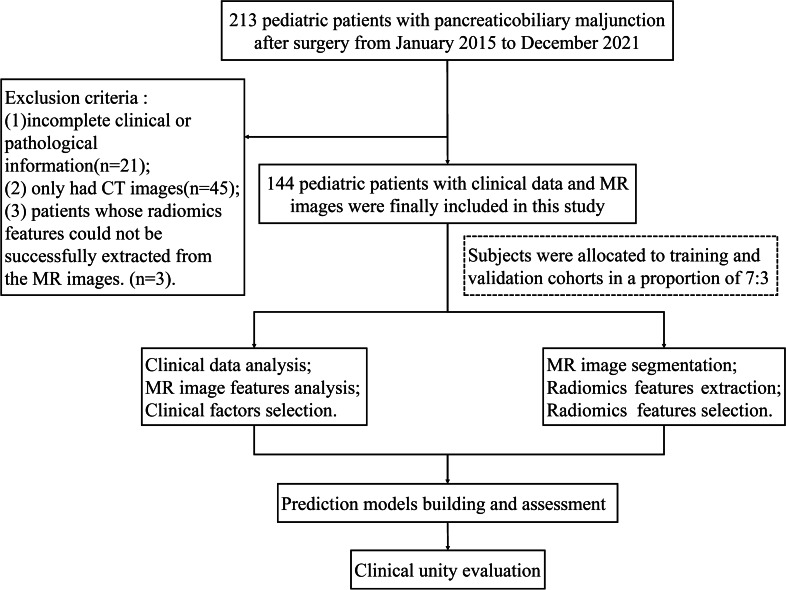
Patient recruitment and study design

The initial screening identified a total of 213 PBM children during a period from January 1, 2015 to December 31, 2021.The inclusion criteria were as follows: (1) possession of pathological results from surgical specimens; (2) completion of surgery within 1 month after MR examination; and (3) availability of complete clinical data. The exclusion criteria were as follows: (1) incomplete clinical or pathological information; (2) patients diagnosed by CT scan alone, without MR scan; or (3) patients whose radiomics features could not be successfully extracted from the MR images. In total, 144 cases were included in the final analysis (Fig. 1).

Due to the small number of cases at Xuzhou Children’s Hospital (n = 26), we did not adopt the conventional approach of using cases from one site as training cohort and cases from the other site for external validation. Instead, the 144 cases were randomly split at a 7:3 ratio to a training and a validation cohort. Clinical features considered as candidate variables for the model included sex, age (in years), abdominal pain, jaundice, fever, vomiting, liver dysfunction, pancreatitis, and elevated white blood cell (WBC) count. Liver dysfunction was defined as an elevation in serum aspartate aminotransferase (AST) and serum alanine aminotransferase (ALT) levels, while pancreatitis was defined as a preoperative serum amylase or lipase level of more than threefold the normal upper limit.

### Image acquisition, segmentation, and feature extraction

**Fig. 2:**
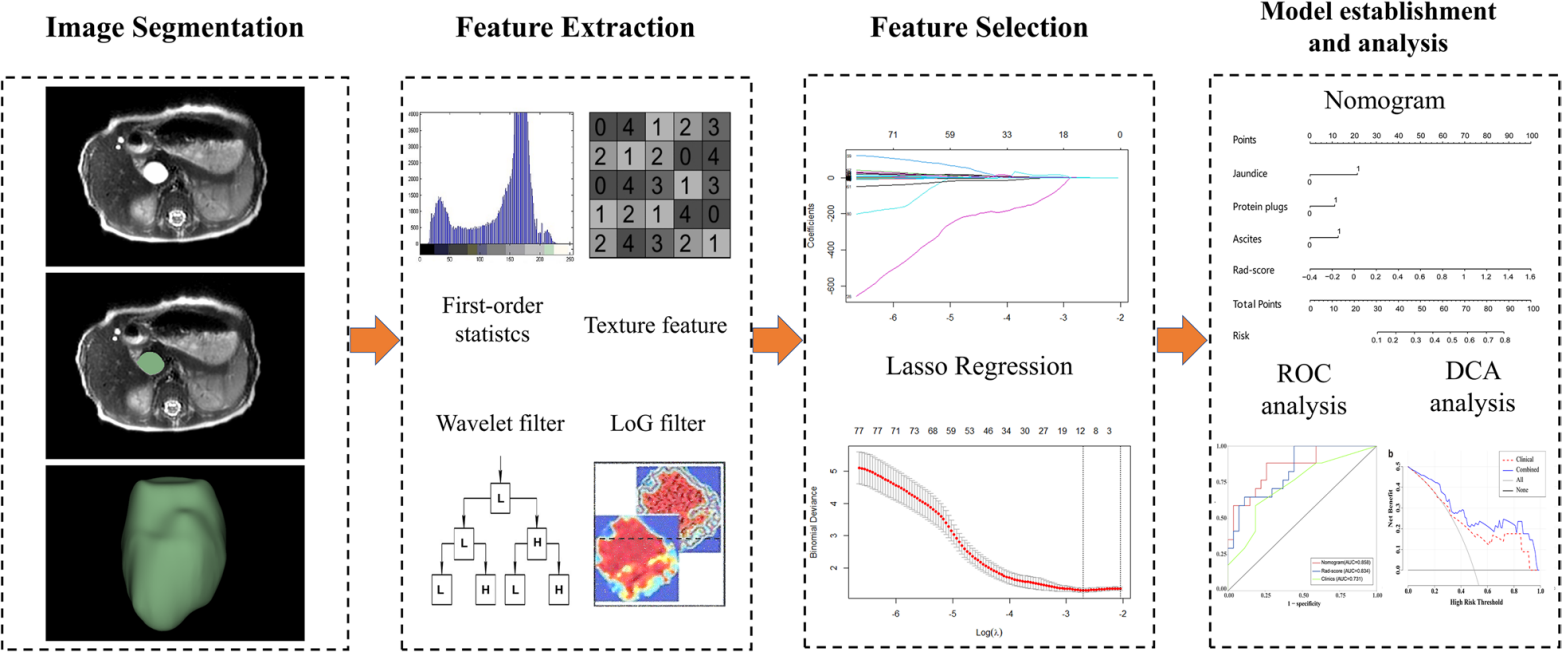
Workflow of the radiomics analysis

All MR images were retrieved from the picture archiving and communication system (PACS) for further analysis. Regions of interest (ROIs) of the T2W images and radiomics feature extraction were performed using 3D Slicer software (version 4.10.2, https://www.slicer.org). The procedure of MR image acquisition, image segmentation, and feature extraction is described in Additional file 1. The radiomic analysis workflow is shown in Fig. 2.

### Imaging analysis

Two pediatric radiologists (L.ZH., with 3 years of experience in pediatric radiology; and Y.Y., with 9 years of experience in pediatric radiology) performed initial analysis of all images. They were blinded to the results of pathological diagnosis of cholangitis. The following MR imaging features of PBM were analyzed: protein plug (present or not), ascites (present or not), Todani classification of congenital biliary dilatation (CBD) (I, IVa), and Komi classification of PBM (I, II, III). Disagreements were resolved by discussion and consensus.

### Selection of clinical variables

Univariate logistic regression was used to screen for demographic and clinical variables. Variables with *p* < 0.1 in the univariate regression were entered into the multivariate regression analysis. Results are shown as odds ratios (ORs) and 95% confidence intervals (CIs).

### Selection of radiomics features and Rad‑score building

All imaging features were normalized using z-score normalization before feature extraction. To minimize the impact of dimensionality, selection of features was conducted in 3 steps using the training cohort. First, inter- and intra-observer analyses were used to assess the features’ reliability and reproducibility [18]; those with ICCs < 0.75 were eliminated from further consideration. Second, features with ICCs > 0.75 were tested using one-way analysis of variance (ANOVA) to select potentially important ones. Finally, LASSO regression then was conducted to eliminate redundant and irrelevant features [19]. Additionally, Spearman correlation coefficients were calculated for the features selected by LASSO to avoid the underlying severe linear dependence. When the value is less than 0.9, we considered that there is no correlation between the selected features [20].

In order to achieve a high and robust performance of classification, three machine learning classifiers, logistic regression (LR), support vector machine (SVM), and decision tree (DT), were implemented. The performances of the radiomics signatures that we developed were then validated for both the training and validation cohorts according to the area under the receiver operator characteristic (ROC) curve. The Delong test was used to compare the performance of three different machine learning classifiers.

To simplify the model, a Rad-score (the sum of the products of the selected features and their corresponding coefficients) was used for subsequent analysis.

### Model development

Diagnostic models were developed based on clinical features alone, Rad-score alone, and clinical features plus Rad-score. Performance of the models (based on the clinical features alone, Rad-score alone, and combined model) was compared using the area under the receiver operator characteristic (ROC) curve. The Delong test was used to compare the performance of three different models. Hosmer–Lemeshow test was used to assess the goodness-of-fit of the models. Decision curve analysis (DCA) was conducted to assess the clinical and combined models through calculating the net benefit at different threshold probabilities.

### Radiomics nomogram building

To provide clinicians with an individualized and easy-to-use tool for the preoperative diagnosis of the occurrence of chronic cholangitis in PBM patients, the combined model was visualized as a radiomics nomogram. A radiomics nomogram score (Nomo-score) was calculated based on the significant clinical features and the Rad-score.

### Statistical analysis

Statistical analysis was performed using SPSS 26.0 software (IBM) and the R programming language (ver. 4.1.2, http://www.r-project.org). Clinical characteristics were measured based on the variable type. The Shapiro–Wilk’s test was employed to assess the normality of the distributions, and homogeneity of variance (homoscedasticity) was assessed using Bartlett’s test. Differences in continuous variables were assessed by t-test or Mann–Whitney U test. Categorical variables were analyzed using Chi-squared or Fisher’s exact-probability testing. The clinical characteristics with a *p* < 0.1 in univariate analysis were included in the multivariate models. The statistical significance level in the final models was set at *p* < 0.05.

LASSO regression was conducted using the “glmnet” package. The “pROC” package was used to plot the ROC curve. The Spearman correlation analysis was performed using the “corrplot” package. Construction of the model that combines clinical variables and radiomics features was carried out using the “rms” package. The Hosmer–Lemeshow test was conducted using the “Resource Selection” package. Decision curves analysis was performed using the “rmda” package.

## Results

### Patient characteristics and clinical features selection

The final analysis included a total 144 children. Pathological examination after surgery showed chronic cholangitis in 56 children. Patients were randomly assigned to the training (n = 100) and validation (n = 44) cohorts at a ratio of 7:3. Their characteristics are detailed in Table 1. There was no significant difference in incidence of cholangitis between training and validation cohorts (0.390 and 0.386, respectively).

**Table 1:** Clinical characteristics in the training and validation cohorts

Variable	Training cohort (*n* = 100)			Validation cohort (*n* = 44)		
	Cholangitis (*n* = 39)	Non-cholangitis (*n* = 61)	*p* value	Cholangitis (*n* = 17)	Non-cholangitis (*n* = 27)	*p* value
*Sex (%)*			0.759			0.559
Male	10 (25.6)	14 (23.0)		4 (23.5)	4 (14.8)	
Female	29 (74.4)	47 (77.0)		13 (76.5)	23 (85.2)	
Age (mean ± SD) (years)	3.90 ± 3.32	3.46 ± 3.19	0.521	3.03 ± 2.61	3.69 ± 3.35	0.497
*Komi classification (%)*			0.112			0.381
Type I	17 (43.6)	28 (45.9)		9 (53.0)	12 (44.5)	
Type II	20 (51.3)	22 (36.1)		8 (47.0)	13 (48.1)	
Type III	2 (0.1)	11 (18.0)		0 (0.0)	2 (7.4)	
*Todani classification (%)#*			0.387			0.353
Type I	22 (56.4)	29 (47.5)		7 (41.2)	15 (55.6)	
Type IVa	17 (43.6)	32 (52.5)		10 (58.8)	12 (44.4)	
*Abdominal pain (%)*			0.442			0.548
Yes	26 (66.7)	36 (59.0)		11 (64.7)	21 (77.8)	
No	13 (33.3)	25 (41.0)		6 (35.3)	6 (22.2)	
*Jaundice (%)*			0.040*			0.020*
Yes	18 (46.2)	16 (26.2)		8 (47.1)	3 (11.1)	
No	21 (53.8)	45 (73.8)		9 (52.9)	24 (88.9)	
*Fever (%)*			1.000			0.167
Yes	5 (12.8)	7 (11.5)		1 (5.9)	5 (18.5)	
No	34 (87.2)	54 (88.5)		16 (94.1)	22 (81.5)	
*Vomiting (%)*			0.098*			0.651
Yes	24 (61.5)	28 (45.9)		10 (9.3)	14 (51.9)	
No	15 (38.5)	33 (54.1)		7 (7.7)	13 (48.1)	
*Protein plug (%)*			0.030*			0.029*
Yes	22 (56.4)	14 (23.0)		10 (58.8)	7 (10.4)	
No	17 (43.6)	47 (77.0)		7 (41.2)	20 (16.6)	
*Ascites (%)*			0.000*			0.353
Yes	33 (84.6)	30 (49.1)		10 (58.8)	12 (44.4)	
No	6 (15.4)	31 (50.9)		7 (41.2)	15 (55.6)	
*Liver dysfunction (%)*			0.353			0.013*
Yes	24 (61.5)	43 (70.5)		15 (88.2)	14 (51.9)	
No	15 (38.5)	18 (29.5)		2 (11.8)	13 (48.1)	
*Pancreatitis (%)*			0.997			0.218
Yes	16 (41.0)	25 (41.0)		5 (29.4)	13 (48.1)	
No	23 (59.0)	36 (59.0)		12 (70.6)	14 (51.9)	
*Elevated WBC count (%)*			0.384			0.429
Yes	12 (30.8)	24 (36.3)		9 (52.9)	11 (40.7)	
No	27 (69.2)	37 (60.7)		8 (47.1)	16 (59.3)	

*p < 0.1 *SD* standard deviation; *WBC* white blood cell

#Almost all individuals with CBD of Todani Type I (except for Type Ib) and Type IVa have associated PBM, but types Ib, II, III, IVb, and V are not accompanied by PBM in almost all cases

In the univariate analysis, chronic cholangitis was associated with jaundice, protein plug, and ascites. In subsequent multivariate LR analysis, chronic cholangitis was independently associated with jaundice (OR = 3.007; 95% CI, 1.362–6.638; *p* = 0.006), protein plug (OR = 3.527; 95% CI, 1.593–7.808; *p* = 0.002), and ascites (OR = 3.793; 95% CI, 1.690–8.513; *p* = 0.001) (Table 2).

**Table 2:** Results of univariate and multivariate logistic analysis

Variable	Univariate analysis		Multivariate analysis	
	OR (95% CI)	*p* value	OR (95% CI)	*p* value
Sex	1.296 (0.585, 2.874)	0.523		
Age	1.010 (0.909, 1.122)	0.851		
Komi classification	0.753 (0.449, 1.264)	0.283		
Todani classification	1.074 (0.549, 2.100)	0.834		
Abdominal pain	1.059 (0.523, 2.144)	0.873		
Jaundice	3.147 (1.516, 6.534)	0.002*	3.007 (1.362, 6.638)	0.006**
Fever	0.760 (0.268, 2.157)	0.606		
Vomiting	1.693 (0.857, 3.341)	0.129		
Protein plug	2.970 (1.449, 6.088)	0.003*	3.527 (1.593, 7.808)	0.002**
Ascites	3.623 (1.714, 7.656)	0.001*	3.793 (1.690, 8.513)	0.001**
Liver dysfunction	1.248 (0.608, 2.559)	0.546		
Pancreatitis	0.789 (0.398, 1.568)	0.499		
Elevated WBC count	0.909 (0.456, 1.810)	0.785		

Intercept = − 2.105; *WBC* white blood cell; *OR* odds ratio; *CI* confidence interval

*p < 0.1

**p < 0.05

### Radiomics feature selection and Rad‑score building

**Fig. 3:**
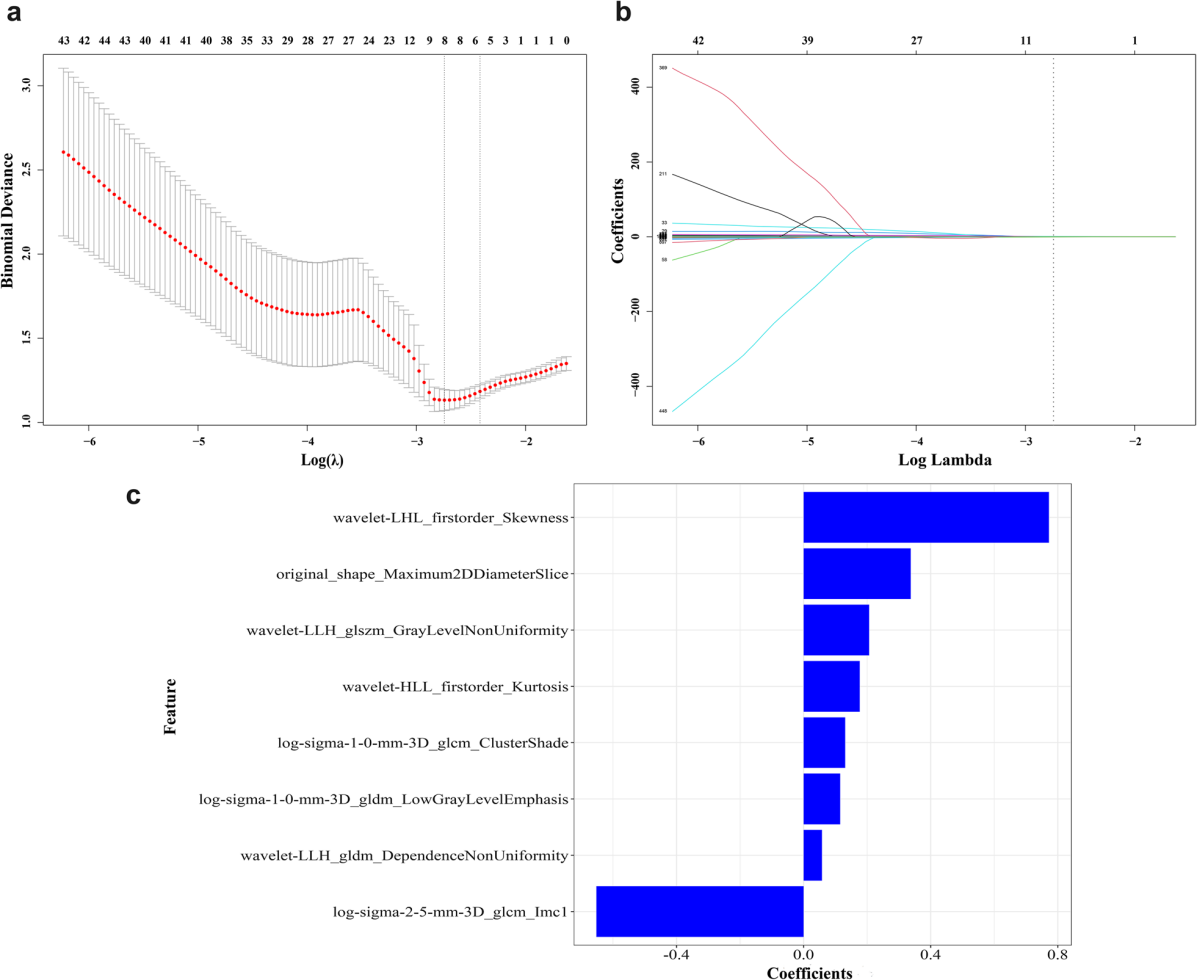
Radiomics feature selection using the least absolute shrinkage and selection operator (LASSO) regression model. **a** Tuning parameter (*λ*) selection in LASSO model used tenfold cross-validation via minimum criterion. The optimal values of the LASSO tuning parameter (*λ*) are indicated by the dotted vertical lines, and a value *λ* of 0.064 with log(*λ*) = − 2.743 was selected. **b** LASSO coefficient profiles of the 1056 radiomics features. A coefficient profile plot was produced versus the log (*λ*) sequence. The dotted vertical line was drawn at the value selected using tenfold cross-validation, in which the selected *λ* resulted in eight nonzero coefficients. **c** The most predictive subset of feature was chosen and the corresponding coefficients were evaluated in the training cohort

Of 1223 extracted radiomics features, 1060 most stable features (both inter-/intra-observer analysis ICC values greater than 0.75) were considered for subsequent analysis. Additional file 1: Fig. S1 displays a figure showing the intra- and inter-class correlation coefficients (ICCs) for radiomics features. After one-way ANOVA, 1056 were retained (*p* < 0.05). These features were then subjected to LASSO analysis to obtain the most valuable ones. We found a best-tuned regularization parameter *λ* of 0.064 under the minimum criteria via tenfold cross-validation. Eventually, eight radiomics features that included four wavelet features, three Laplacian of Gaussian (LoG) features, and one shape feature were nominated to construct the radiomics signature (Fig. 3a, b). The contribution of the radiomics signature is shown in Fig. 3c. In addition, Spearman correlation coefficients among the 8 features ranged from − 0.38 to 0.84, which indicates that there is no collinearity.

The classification performances of the three ML classifiers are presented in Table 3. In the training cohort, AUCs for the LR, SVM, and DT classifiers were 0.896, 0.937, and 0.817, respectively; and corresponding AUCs for the validation cohort were 0.878, 0.847, and 0.719, respectively. In DeLong test, the LR and SVM classifiers were superior to the DT classifier, but overfitting is apparent in the SVM classifier. Accordingly, the LR classifier was chosen for subsequent use. The following Rad-score was derived based on the coefficients weighted by LASSO-logistic regression:

**Table 3:** Comparison of different machine learning classifiers in the training and validation cohorts

Classifiers	Training cohort (*N* = 100)					Validation cohort (*N* = 44)				
	AUC (95% CI)	ACC	SEN	SPE	Delong	AUC (95% CI)	ACC	SEN	SPE	Delong
LR	0.896 (0.826–0.967)	0.876	0.846	0.885	0.019#	0.878 (0.771–0.985)	0.841	0.852	0.824	0.556#
SVM	0.937 (0.877–0.997)	0.920	0.897	0.984	0.001##	0.847 (0.732–0.963)	0.818	0.941	0.741	0.106##
DT	0.817 (0.731–0.902)	0.810	0.615	0.934	0.025###	0.719 (0.516–0.972)	0.750	0.412	1.000	0.025###

*LR* logistic regression; *SVM* support vector machine; *DT* decision tree; *SEN* sensitivity; *SPE* specificity; *ACC* accuracy; *AUC* area under the curve; *CI* confidence interval

#LR versus SVM

##SVM versus DT

###DT versus LR

0.337 × original_shape_Maximum2DDiameterSlice

 + 0.115 × log-sigma-1–0-mm-3D_gldm_LowGrayLevelEmphasis.

 + 0.131 × log-sigma-1–0-mm-3D_glcm_ClusterShade.

– 0.652 × log-sigma-2–5-mm-3D_glcm_Imc1.

 + 0.177 × wavelet-HLL_firstorder_Kurtosis.

 + 0.772 × wavelet-LHL_firstorder_Skewness.

 + 0.058 × wavelet-LLH_gldm_DependenceNonUniformity.

 + 0.206 × wavelet-LLH_glszm_GrayLevelNonUniformity – 1.636.

The Rad-score was significantly higher in the children with vs without chronic cholangitis in both the training (1.752 ± 0.583 vs. 0.832 ± 0.731) and validation cohorts (1.885 ± 0.743 vs. 0.897 ± 0.573) (*p* < 0.001 for both). Additional file 1: Fig. S2 displays a figure showing the Rad-score in the both cohorts.

### Model performance

**Fig. 4:**
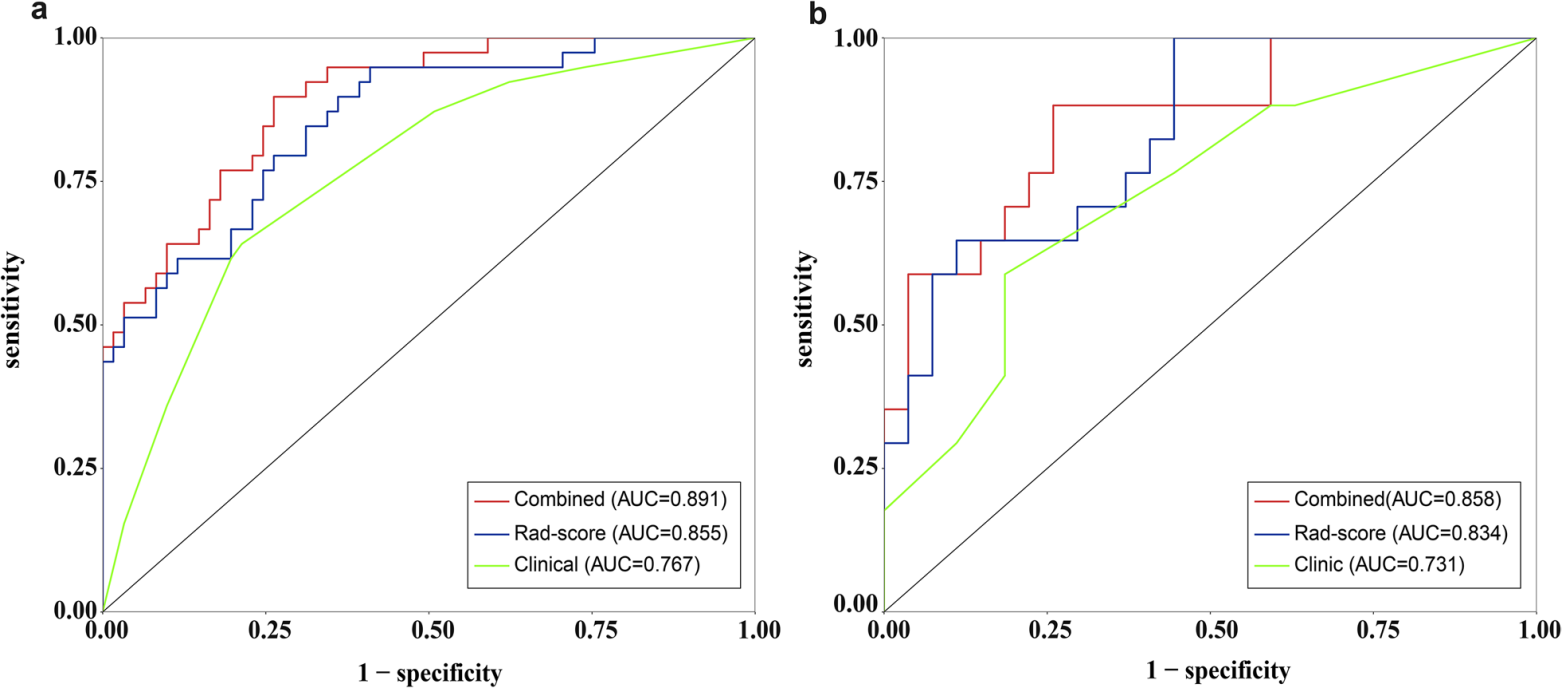
The ROC curves of the three models in the training (**a**) and validation (**b**) cohorts

Performance of the models based on the clinical features alone, Rad-score alone, and both is presented in Table 4 and Fig. 4. The AUC in the validation cohort was 0.731 (95% CI = 0.577–0.885) for the model based on clinical variables alone, 0.834 (95% CI = 0.716–0.953) for the model based on Rad-score only, and 0.858 (95% CI, 0.745–0.972) based on the combined model. In the Delong test, the combined model outperformed the clinical model in the validation cohort (*p* = 0.028).

**Table 4:** Performance of the models based on clinical features alone, Rad-score alone, and both

Models	Training cohort (*N* = 100)					Validation cohort (*N* = 44)				
	AUC (95% CI)	ACC	SEN	SPE	Delong	AUC (95% CI)	ACC	SEN	SPE	Delong
Clinical	0.767 (0.673–0.860)	0.743	0.641	0.787	0.138#	0.731 (0.577–0.885)	0.718	0.588	0.815	0.251#
Rad-score	0.855 (0.781–0.929)	0.735	0.949	0.590	0.136##	0.834 (0.716–0.953)	0.727	1	0.556	0.577##
Combined	0.891 (0.830–0.952)	0.805	0.897	0.738	0.002###	0.858 (0.745–0.972)	0.814	0.882	0.741	0.028###

*AUC* area under the curve; *CI* confidence interval; *ACC* accuracy; *SEN* sensitivity; *SPE* specificity

#Clinical versus Rad-score

##Rad-score versus combined

###Combined versus clinical

**Fig. 5:**
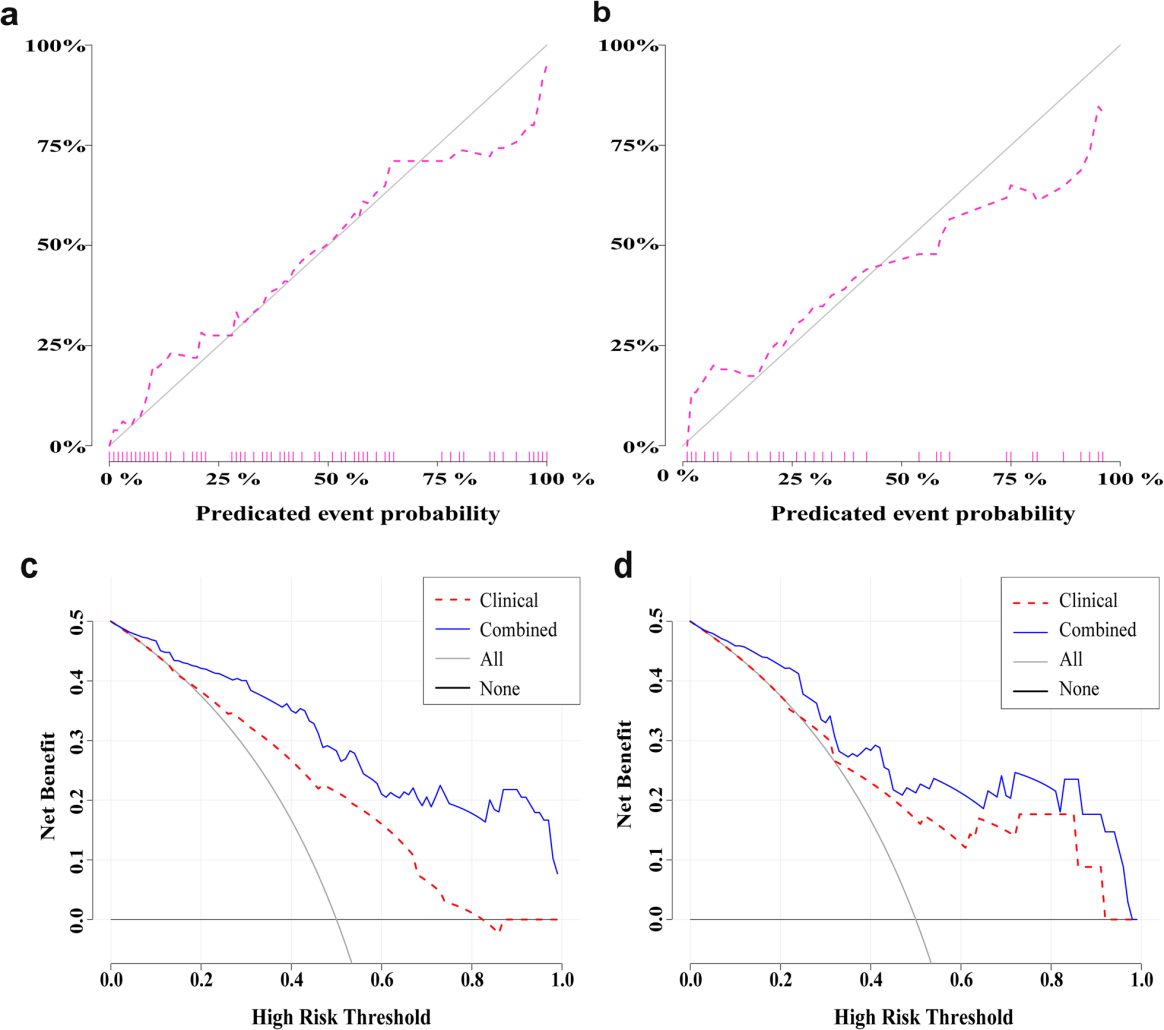
Calibration curves of the combined model in the training (**a**) cohort and validation (**b**) cohort. The combined model predicted the incidence of cholangitis and actual rate are, respectively, plotted on the x- and y-axis. The diagonal line represents a faultless calculation of an ideal model. Pink lines represent outcomes of the combined model in training and test cohort. A closer lining to the diagonal line indicates a more accurate calculation. The decision curve analysis (DCA) of the clinical model (red line) and the combined model (blue line) in the training (**c**) cohort and validation (**d**) cohort. The y-axis indicates the net benefit; x-axis indicates threshold probability. The gray line represents the decision curve of the assumption that all PBM cases with cholangitis, and the black line shows the decision curve of the assumption that no PBM case with cholangitis. The DCA revealed that the combined model was more advantageous than the clinical model

The combined model had 0.814 accuracy, 0.882 sensitivity, and 0.741 specificity. A calibration curve analysis revealed good agreement between the predicted and actual probabilities in diagnosing chronic cholangitis in the training and validation cohorts (*p* = 0.977 and 0.370 in Hosmer–Lemeshow test; Fig. 5a, b). DCA demonstrated higher overall net benefit with the combined model than the clinical model (Fig. 5c, d).

### Development of a radiomics nomogram

**Fig. 6:**
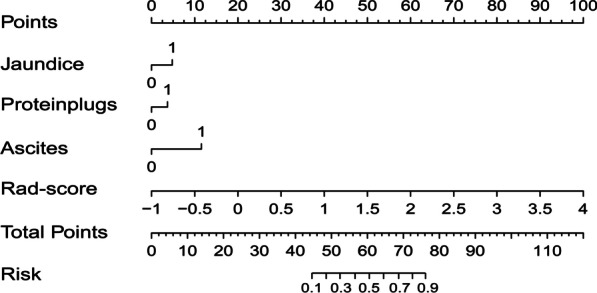
A radiomics nomogram combined the Rad‑score and the selected clinical factors

We developed a radiomics nomogram for diagnosis of chronic cholangitis in PBM children (Fig. 6). The specific formula is:

$$0.851 \times \text{jaundice} \, + \, 0.593 \times \text{protein plug} \, + 1.234 \times \text{ascites} \, + \, 2.459 \times \text{Rad - score} \, {-} \, 4.995$$

## Discussion

The results from the current study showed that adding radiomics features in combination to clinical variables could enhance the accuracy of diagnosing chronic cholangitis in children with PBM. A strength of the study was the development of a single Rad-score that summarize a variety of relevant radiomics features, and thus convenience for use in clinical practice. The AUC under the ROC of the combined model was 0.858, with 0.882 sensitivity and 0.741 specificity. Furthermore, the calibration curves and DCA illustrated the clinical utility of this nomogram.

The biliary wall in PBM patients usually under prolonged stimuli from chronic inflammation [21]. Chronic cholangitis not only increases intraoperative complications in PBM patients (*e.g.*, bleeding at the site of resection) but is also correlated with malignancy in the long term [1, 11, 22]. Early interventions for cholangitis in PBM patients can have great effects on short- and long-term outcomes. Accordingly, establishing a method that accurately identifies cholangitis in PBM patients is of paramount importance.

Currently, five primary tools are used in clinical practice to diagnose cholangitis: US, CT, MRCP, endoscopic retrograde cholangiography (ERCP), and endoscopic ultrasonography (EUS) [8]. Each of these modalities has its advantages and limitations. For example, although transabdominal US can be used to diagnose cholangitis by observation of bile duct wall thickness (> 0.8 mm), dilated bile ducts will lead to diminished diagnostic performance of US [8]. MRCP is the first choice in diagnosing biliary disorders, but it is easily affected by motion artifacts. It is difficult to depict fine structures such as the pancreaticobiliary anatomy in pediatric patients at MRCP, especially in babies and toddlers [2].MRCP may also miss small stones and bile duct dilatations [9]. ERCP is the reference standard for diagnosis of cholangitis [9]. However, it is an invasive surgery that may induce various complications, such as pancreatitis, cholangitis, bleeding, infection, and thus not be widely used in pediatric cases [2]. Therefore, the use of these traditional tools typically presents a significant technical challenge in the diagnosis of biliary disorders.

Compared with conventional imaging modalities, radiomics allows the detection of many subtle changes that are not detectable by manual visual assessment and facilitates high-throughput extraction of quantitative data from images. Such data are more reflective of quantitative information drawn from images than are those assessed by the naked eye. Therefore, radiomics can clarify an underlying biological condition and shows robust predictive power [23, 24]. Radiomics has been increasingly applied to the study of non-oncological diseases. For example, in a recent study on inflammatory lesions of the intestinal tract, Zhu et al. [25] improved predictive performance so as to distinguish Crohn disease from intestinal tuberculosis by creating a predictive model that combined clinical factors with radiomics features; we used this model as a reference for our study.

The established radiomics signature in the present study consisted of four wavelet features, three LoG features and one shape feature showed favorable performance. Seven out of the 8 selected features were deep features. This is consistent with the fact that deep features reflect higher-order imaging patterns and capture more imaging heterogeneity than low-level shape, intensity, and texture features. Recent studies focused on other organs such as the liver and intestines have shown a potential correlation between tissue fibrosis and radiomics features [26, 27], and it is therefore quite reasonable to assume that the radiomics signature constructed in this study might reflect fibrosis in the biliary epithelia [28]. However, generating a map that encompasses radiomics heterogeneity and pathological characteristics of chronic inflammation in the bile duct (e.g., hyperemia, edema, inflammatory infiltration) remains challenging and requires further study.

*High*-*dimensional features* may make the possibility of overfitting [29]. Although 1223 radiomics features were derived from the T2WI, only 1060 were extracted for further feature selection because they are demonstrated to have a good intra- and inter-correlation coefficient. Firstly, ANOVA test was utilized to optimize the feature set. Then, we proceeded to feature selection and modeling by using LASSO regression. Finally, Spearman correlations were computed to preclude the selected features multicollinearity. Moreover, we also compared the performance of radiomics models developed by three common machine learning classifiers. With implementation of the excellent feature selection methods and the machine learning classifier, the final radiomics model demonstrated comparable diagnostic performance in training and validation cohorts, indicating that the strategy in the present study effectively mitigated the overfitting issues.

Three of the clinical characteristics were included in the radiomics nomogram: jaundice, protein plug, and ascites. Jaundice and protein plug are established markers of cholangitis [8, 30, 31]. In contrast to previous studies of acute cholangitis [9, 32], ascites was associated with chronic cholangitis and thus included in the diagnostic model. Such a discrepancy reflects the distinct focus on chronic cholangitis in the current study and acute cholangitis in previous studies. However, the reason for ascites associated with chronic cholangitis should be further studied in future.

The sensitivity and accuracy of the combined model (0.882 and 0.814, respectively) were higher than those of the clinical model. The AUC under the ROC was 0.858 in the validation cohorts. The calibration curves demonstrated good consistency between the predicted value and the actual outcome. Furthermore, DCA results revealed that the combined model had more net benefits than those of the clinical model at different threshold probabilities.

The proposed nomogram can aid individual preoperative risk assessment, which might help surgeons to select a reasonable surgical modality for patients suspected of having a higher risk of chronic cholangitis. Furthermore, more active follow-up should be carried out after surgery for these patients to prevent postoperative complications. Therefore, using an MRI-based radiomics nomogram can be regarded as a promising assistive tool in preoperative prediction of chronic cholangitis risk in pediatric patients with PBM.

There are several key limitations in the present study. First, the results are subject to a variety of biases due to the retrospective nature of the study. Second, the radiomics features were limited to T2-weighted MRI images. Whether multiparametric MRI (including contrast-enhanced MR images) are more useful is unknown. Third, the formal pathological classification of choledochal cysts is not well established yet; thus, we could not examine in detail the relationship between the severity of chronic cholangitis and the treatment and prognosis of PBM patients[33]. Finally, manual segmentation of ROIs was time-consuming and may have introduced significant bias due to a partial-volume effect.

In conclusion, a model that combines key clinical variables and radiomics signature is helpful in the diagnosis of chronic cholangitis in PBM children. The results from the current study also indicate that it is possible to simplify complex radiomics features into a single Rad-score for use in daily practice.

## Supplementary Information

**Additional file 1**. Supplementary materials on image acquisition, image segmentation, feature extraction and additional figures.

## Data Availability

The datasets generated and analyzed during the current study are not publicly available due to local restrictions of data protection but are available from the corresponding author on reasonable request.

## Abbreviations

ACC: Accuracy
AUC: Area under the curve
CBD: Congenital biliary dilatation
CI: Confidence interval
CT: Computed tomography
DCA: Decision curve analysis
ERCP: Endoscopic retrograde cholangiography
ICCs: Inter-/intra-observer class correlation coefficients
LASSO: Least absolute shrinkage and selection operator
LoG: Laplacian of Gaussian
LR: Logistic regression
ML: Machine learning
MRCP: Magnetic resonance cholangiopancreatography
MRI: Mresonance imaging
OR: Odds ratio
PACS: Picture archiving and communication system
PBM: Pancreaticobiliary maljunction
Rad-score: Radiomics score
ROC: Receiver operating characteristic
SVM: Support vector machine
T2WI: T2-weighted imaging
US: Ultrasonography
WBC: White blood cell
