# Supplementary material for: Development of a simplified model and nomogram in preoperative diagnosis of pediatric chronic cholangitis with pancreaticobiliary maljunction using clinical variables and MRI radiomics
Source: Insights Imaging. 2023 Mar 8;14:41. doi: 10.1186/s13244-023-01383-z (PMC9992494; doi:10.1186/s13244-023-01383-z)

ELECTRONIC SUPPLEMENTARY MATERIAL

Development of a Simplified Model and Nomogram in Preoperative Diagnosis of Paediatric Chronic Cholangitis With Pancreaticobiliary Maljunction Using Clinical Variables and MRI Radiomics

1. Image acquisition

2. Image segmentation

3. Feature extraction

4. Supplementary Figure 1. Histogram of the intra- and inter-class correlation coefficient (ICCs) for radiomic features.

5. Supplementary Figure 2. The distribution of the Rad-score in the training and validation cohorts.

1. Image acquisition

MR scans were conducted using 2 3.0-T MRI scanners (Discovery MR750w; GE Healthcare, Milwaukee, WI, USA; and Discovery MR750; GE Healthcare, Piscataway, NJ, USA), both having 32-channel body coils. Children who could not remain motionless during examinations were sedated with 0.5 mg/kg chloral hydrate.

The MR protocols and imaging parameters were as follows:(1) The axial gradient echo (GRE) T1-weighted imaging (T1WI) with a repetition time (T_R)/echo time (T_E) ratio of 6–10/2–8 ms, flip angle of 15 degrees, slice thickness of 4–5 mm, a slice gap of 4–5 mm, matrix of 256–320 × 256–320, and a field of vision (FOV) of 30×30 cm; (2) The axial fast spin-echo (FSE) T2-weighted imaging (T2WI) with a T_R/T_E ratio of 3800-

6500/65-80 ms, slice thickness of 4–5 mm, a slice gap of 4–5 mm, matrix of 256–320 × 256–320, and an FOV of 30×30 cm; (3) The coronal single-shot fast-spin echo(SSFSE) T2WI with a T_R/T_E ratio of 1800–2200/60–70 ms, slice thickness of 3 mm, a slice gap of 3 mm, matrix of 256–320 × 256–320, and an FOV of 30×30 cm; and (4) the respiration-triggered coronal three-dimensional MRCP (RTr Cor 3D MRCP) with a T_R/T_E ratio of 4000–6000/800–1000ms, slice thickness of 2–3 mm, a slice gap of 1–1.5 mm, number of excitations (NEX) at 1, and an FOV of 30×30 cm. We selected T2WIs in Digital Communications in Medicine (DICOM) format for further analysis.

2. Image segmentation

Regions of interest (ROIs) were manually segmented using 3D Slicer software (version 4.10.2; https://www.slicer.org) by the two radiologists, who were blinded to histopathological data. Lesion outlines were traced on all contiguous slices to generate three-dimensional ROIs.

To test feature stability, readers 1 and 2 extracted radiomics features from 50 randomly chosen patients, and reader 1 then repeated the same procedure 2 weeks later. We calculated inter-/intra-observer class correlation coefficients (ICCs) to evaluate the consistency and reproducibility of the generated features. Features with ICC > 0.75 in both intra- and inter-observer agreement analyses were then included in subsequent analyses. Reader 1 segmented the ROIs for the remaining images.

3. Feature extraction

To correct the different pixel spacing of the MR image volumes for patients in two institutions, all images were resampled to 1 × 1 × 1 mm^3 voxels and their intensity

range was normalized to 0 to 255. Then, feature extraction was conducted using the radiomics module in 3D Slicer 4.10.2 platform.

A total of 1223 radiomic features were ultimately extracted. The features can be categorized into the following 4 groups:

(1) Shape-based features (14 features): these features describe three-dimensional size and shape of the region of interest (ROI), such as volume, surface area, and diameter.

(2) First-order statistics features (18 features): these features are related to the gray tone distribution of the pixel intensity and mainly used to perform first-order statistics like means, standard deviation, kurtosis, skewness, uniformity, energy and entropy.

(3) Texture features (75 features): these features are derived from several gray-level matrix and mainly describe the spatial relationship between pixels. The gray-level matrix included gray level co- occurrence matrix (GLCM; 24 features), gray level run length matrix (GLRLM;16 features), gray level size zone matrix (GLSZM;16 features), neighboring gray tone difference matrix (NGTDM;5 features) and gray level dependence matrix (GLDM;14 features).

(4) LoG features (372 features) and Wavelet features (744 features):to enhance intricate patterns in the data invisible to the human eye, advanced filters, including Laplacian of Gaussian (LoG; sigma, 1.0, 1.5, 2.0 and 2.5 mm), and wavelet decompositions with all possible combinations of high (H) or low (L) pass filter in each of the three dimensions (HHH, HHL, HLH, LHH, LLL, LLH, LHL, HLL), were applied.

Thus, (18+75) ×4 LoG features and (18+75) ×8 wavelet features were obtained from the LoG transform and wavelet transform, respectively.

The total number of radiomic features could be calculated as:

$$14 + 18 + 75 + (18 + 75) \times 4 + (18 + 75) \times 8 = 1223$$

4. Supplementary Figure 1. Histogram of the intra- and inter-class correlation coefficients (ICCs) for radiomics features derived from MR imaging.

After reproducible analysis, 1116 (a) and 1075 (b) features were included. Finally, 1060 most stable features (both intra- and inter-class correlation coefficient values greater than 0.75) were considered for subsequent analysis.

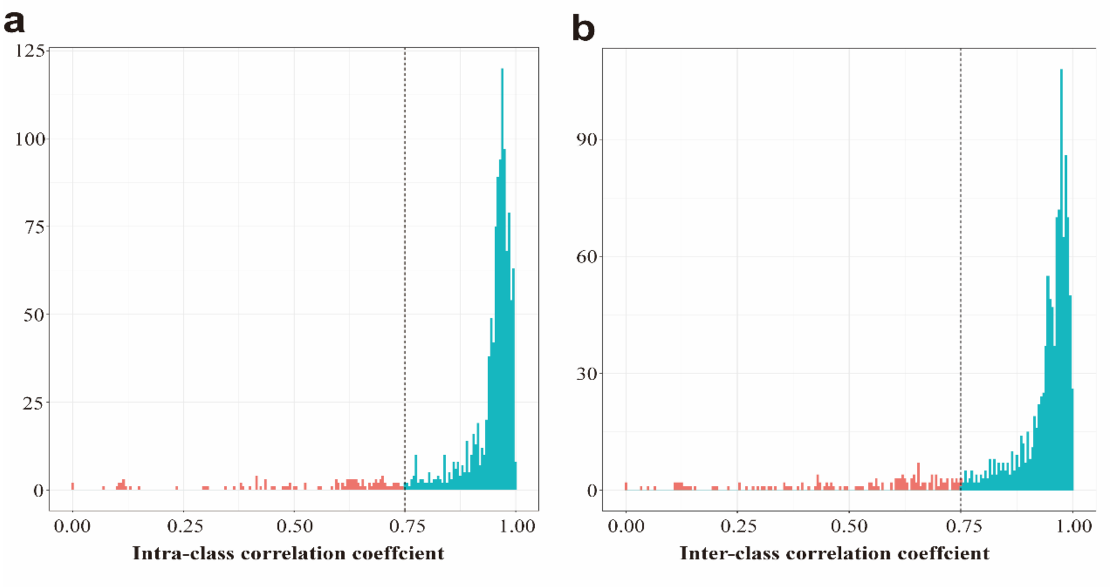

5. Supplementary Figure 2. Violin plot of the Rad-score in the training and validation cohorts. The longitudinal length of the violin plot indicates the range for the score in cholangitis and non-cholangitis groups, while the transverse width indicates the frequency for the score.

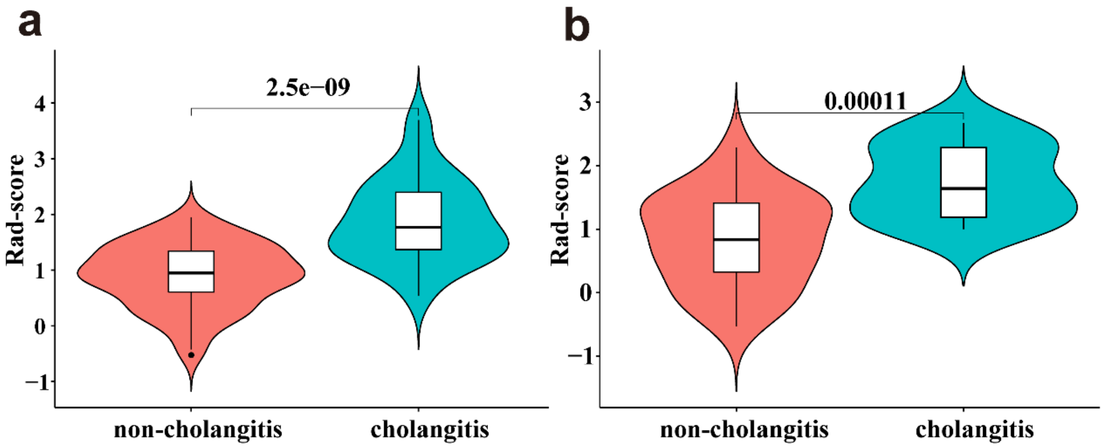

Supplement: Supplementary file 1 — Additional file 1. Supplementary materials on image acquisition, image segmentation, feature extraction and additional figures. [file 13244_2023_1383_MOESM1_ESM.pdf]
